# CAGE profiling of ncRNAs in hepatocellular carcinoma reveals widespread activation of retroviral LTR promoters in virus-induced tumors

**DOI:** 10.1101/gr.191031.115

**Published:** 2015-12

**Authors:** Kosuke Hashimoto, Ana Maria Suzuki, Alexandre Dos Santos, Christophe Desterke, Agnese Collino, Serena Ghisletti, Emilie Braun, Alessandro Bonetti, Alexandre Fort, Xian-Yang Qin, Enrico Radaelli, Bogumil Kaczkowski, Alistair R.R. Forrest, Soichi Kojima, Didier Samuel, Gioacchino Natoli, Marie Annick Buendia, Jamila Faivre, Piero Carninci

**Affiliations:** 1 RIKEN Center for Life Science Technologies, Division of Genomic Technologies, Yokohama, Kanagawa, 230-0045 Japan;; 2 INSERM, U1193, Paul-Brousse Hospital, Hepatobiliary Centre, 94800 Villejuif, France;; 3 Université Paris Saclay, Faculté de Médecine Le Kremlin Bicêtre, 94800 Villejuif, France;; 4 European Institute of Oncology (IEO), Department of Experimental Oncology, IFOM-IEO Campus, 20139 Milan, Italy;; 5 RIKEN Center for Life Science Technologies, Division of Bio-function Dynamics Imaging, Wako, Saitama, 351-0198, Japan;; 6 VIB Center for the Biology of Disease, KU Leuven Center for Human Genetics, B-3000 Leuven, Belgium;; 7 Assistance Publique-Hôpitaux de Paris (AP-HP), Pôle de Biologie Médicale, Paul-Brousse Hospital, 94800 Villejuif, France

## Abstract

An increasing number of noncoding RNAs (ncRNAs) have been implicated in various human diseases including cancer; however, the ncRNA transcriptome of hepatocellular carcinoma (HCC) is largely unexplored. We used CAGE to map transcription start sites across various types of human and mouse HCCs with emphasis on ncRNAs distant from protein-coding genes. Here, we report that retroviral LTR promoters, expressed in healthy tissues such as testis and placenta but not liver, are widely activated in liver tumors. Despite HCC heterogeneity, a subset of LTR-derived ncRNAs were more than 10-fold up-regulated in the vast majority of samples. HCCs with a high LTR activity mostly had a viral etiology, were less differentiated, and showed higher risk of recurrence. ChIP-seq data show that MYC and MAX are associated with ncRNA deregulation. Globally, CAGE enabled us to build a mammalian promoter map for HCC, which uncovers a new layer of complexity in HCC genomics.

Hepatocellular carcinoma (HCC) accounts for 70%–85% of total liver cancers. The major risk factors of HCC are chronic HBV and HCV infections and alcohol (Jemal et al. 2011). The development of HCC is a heterogeneous multistep process associated with genetic alteration and dysregulation of gene expression. Recent whole-genome studies in HCC identified tens of thousands of somatic mutations, several of which occur in chromatin regulators, suggesting that the transcriptional network might have been disrupted through reorganization of chromatin structure (Totoki et al. 2011; Fujimoto et al. 2012). Genome-wide analyses of gene expression in human HCC have identified overexpressed genes (Ladeiro et al. 2008), activated pathways (Huang et al. 2011), and subtypes of HCC (Boyault et al. 2007). However, most of the studies focused on protein-coding genes or microRNAs, and the repertoire of long ncRNAs in HCC tumor tissues remains largely unexplored.

Comprehensive transcriptome studies have revealed that a large proportion of mammalian genomes, including transposable elements (TEs), are transcribed (Kapranov et al. 2002, 2007; Okazaki et al. 2002; Carninci et al. 2005; Cheng et al. 2005; Faulkner et al. 2009; Djebali et al. 2012). LTR retroposons are a major class of TEs, accounting for 8% of the human genome (Lander et al. 2001). The vast majority of long terminal repeat (LTR) retroposons have lost their internal-domain encoding genes and reside as solitary LTRs lacking the ability to retrotranspose (Kovalskaya et al. 2006). Nevertheless, regulatory sequences including promoters and transcription factor binding sites are widely observed within LTR elements (Bourque et al. 2008). We have recently shown that LTRs are massively transcribed in embryonic stem cells and iPS cells, some of which are involved in the maintenance of pluripotency (Fort et al. 2014). Other studies demonstrated a high activity of distinct LTR subfamilies in stem cells using several different methods, including RNA-seq (Kelley and Rinn 2012; St Laurent et al. 2013), DNase-seq (Jacques et al. 2013), and MeDIP-seq (Xie et al. 2013). Furthermore, an appropriate activation of LTRs is essential for iPS reprogramming (Lu et al. 2014; Ohnuki et al. 2014), suggesting that the expression of LTRs might be associated with cancerous features, such as poor differentiation and high proliferation potency.

The Cap Analysis of Gene Expression (CAGE) method has been widely used to identify transcription start sites (TSSs) of ncRNAs and messenger RNAs by capturing the capped 5′ ends of the RNAs. The reproducibility of CAGE for expression measurements has been demonstrated through many studies, including FANTOM (The FANTOM Consortium et al. 2014) and ENCODE (Djebali et al. 2012), discovering a large number of novel ncRNAs and active enhancers. Here, we report the ncRNA transcriptome of human and *Mdr2* knockout (KO) mouse HCC using CAGE, with special emphasis on ncRNAs distant from protein-coding genes. We show that a large proportion of the distal ncRNAs are LTR-derived in human and mouse HCC genomes. The CAGE data revealed three well-defined subclasses of human HCCs corresponding to high, intermediate, and low expression levels of a selected set of LTR ncRNAs, respectively. The LTR-high subclass was correlated with definite clinical features (viral etiology, less differentiated tumors, high risk of recurrence) and MYC pathway activation. ChIP-seq data indicate an active role for transcription factors such as MYC-based complexes in the deregulation of LTR-ncRNAs in HCC.

## Results

### Specific features of distal ncRNAs in HCC transcriptome

We sequenced CAGE libraries for 50 HCC tumor tissues and 50 matched nontumor (NT) tissues from patients with various etiologies, mostly HBV, HCV, and alcohol abuse. We also prepared samples from morphologically normal liver tissues collected at a distance from a liver metastasis of colon cancer in five patients. These last five samples are referred to as “normal” as opposed to the “nontumor” and control for gene signatures affected in morphologically normal liver tissue by nearby inflammation and virus activities. The total number of uniquely mapped reads is 1.7 billion—an average “mapped read count” of 16.0 million for tumors, 15.9 million for nontumors, and 19.5 million for normal tissues (Supplemental Table S1). CAGE peaks (corresponding to TSSs) were determined based on the 5′ position of sequenced reads using Paraclu (Frith et al. 2008).

**Figure 1.**
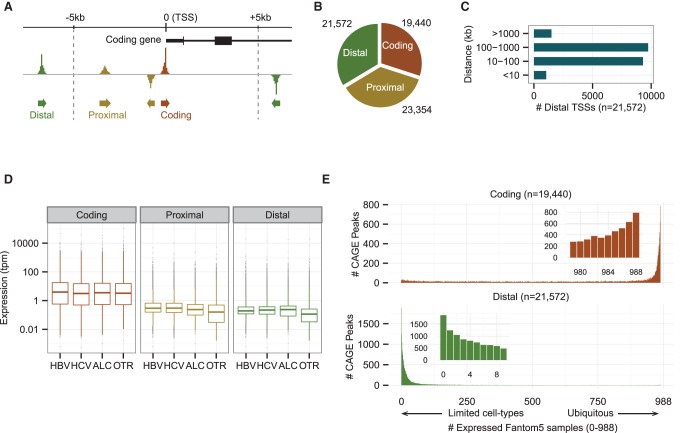
The transcriptome of HCC tumors contains a large number of distal noncoding transcripts. (*A*) Schematic representation of the coding, proximal, and distal CAGE peaks. (*B*) Numbers of peaks in the different groups. (*C*) Distribution of distances between distal peaks and their closest coding peaks. (*D*) Expression levels in the different groups for the indicated etiologies. The data are means over tumors belonging to a given etiology group (tpm: tags per million + 0.001). (*E*) Expression patterns of coding and distal peaks across human body (988 human expression data from the FANTOM5 project). We counted the numbers of tags located within each TSS region in each sample, and then the number of samples that expressed tumor transcripts with the criterion of at least one tag. Coding peaks are ubiquitously expressed, whereas distal peaks are only expressed in limited cell types. Proximal and distal peaks show a similar expression pattern (Supplemental Fig. S1D).

We identified 64,366 distinct CAGE peaks as the transcriptome of human HCC tumor tissues. All the peaks fulfill the following two criteria, as used previously (Fort et al. 2014): The expression level should exceed one tag per million (tpm) in at least one tumor sample; and the peak should be expressed in at least two tumor samples. Each individual peak represents a transcription initiation region at a median length of 45 bp covering 0.17% of the human genome. To distinguish potential ncRNAs from protein-coding genes, we classified the 64,366 peaks identified into three groups with the following distinctive features according to the GENCODE12 annotation (Harrow et al. 2012): (1) a coding peak, which overlaps with a protein-coding TSS (±100 bp) in sense orientation; (2) a proximal peak, which is located within 5 kb of a coding peak or overlaps with a coding exon; and (3) a distal peak, which could not be assigned as either a coding nor a proximal peak (Fig. 1A). It can be assumed that distal peaks are mostly ncRNAs, whereas proximal peaks are mixtures of ncRNAs and unannotated alternative promoters of protein-coding genes. Distal peaks account for 33.5% (21,572/64,366) of the tumor transcriptome (Fig. 1B); half of them are located far (at >100 kb) from expressed coding peaks (Fig. 1C). On the other hand, proximal peaks are enriched in the upstream of coding peaks, which are likely to be products of bidirectional transcription (Supplemental Fig. S1A; Katayama et al. 2005). Expression levels of proximal peaks and their closest coding peaks tend to be positively correlated, whereas those of distal peaks and their closest coding peaks are not correlated, suggesting that distal peaks are not necessarily associated with their neighboring protein-coding genes (Supplemental Fig. S1B). One of the characteristics of ncRNAs is a lower expression level compared to coding RNAs, except for some massively transcribed ncRNAs such as *MALAT1*. Indeed, median expression of proximal and distal transcripts are ∼20–30 times lower than coding transcripts in all the HCCs tumors, irrespective of the etiology (Fig. 1D). Despite their lower level of expression, 90% of the distal peaks are represented by a total of at least 50 reads, identified cumulatively across the collection of samples studied here, and are expressed in more than 10 tumor samples, indicating that the transcripts were not sample-dependent but common to most HCC tumors (Supplemental Fig. S1C).

To estimate how widely the HCC transcripts are expressed in the human body, we used the FANTOM5 atlas, which provides expression data measured by CAGE across a broad panel of primary cells, cell lines, and tissues, including normal adult and fetal liver tissues (The FANTOM Consortium et al. 2014). The distributions of expressed sample counts are strikingly different between coding and distal transcripts (Fig. 1E; Supplemental Fig. S1D). About 80% of coding transcripts are expressed in more than half of the FANTOM5 samples, with the highest peak (mode) at 988 samples, whereas ∼94% of distal transcripts are detected in less than half of the FANTOM5 samples, with the highest peak at 0 samples, indicating that the distal transcripts are expressed in a limited number of tissues and cell linages.

### Aberrant activation of LTR retroviral promoters in HCC tumors

**Figure 2.**
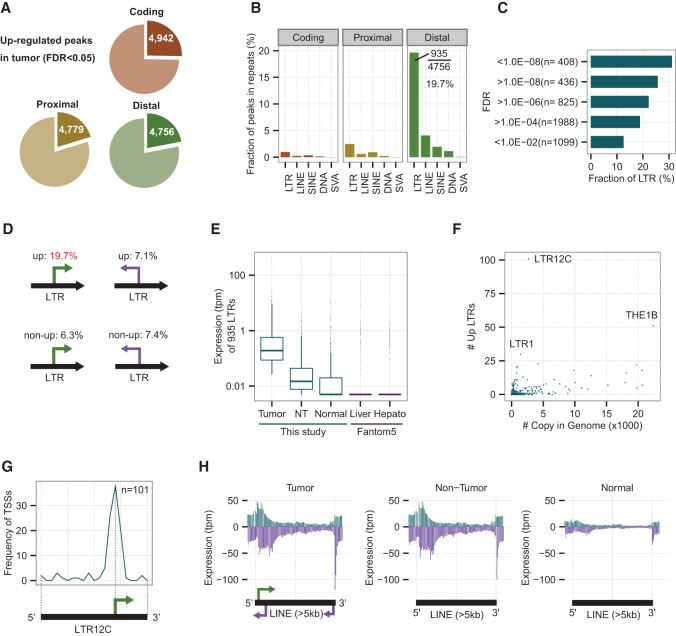
LTR retroviral promoters are up-regulated in human HCC tumors. (*A*) Fractions of significantly up-regulated coding, proximal, and distal peaks in HCC tumors. The threshold is set to *P* < 0.05 (FDR, calculated with edgeR). (*B*) Fractions of peaks overlapping with repetitive elements in the sense direction. Nearly 20% (935/4756) of the up-regulated distal peaks overlap with LTR elements. (*C*) Fractions of peaks overlapping with LTRs at different levels of significance. The most significant bin (FDR < 1.0 × 10^−8^) shows the highest fraction (30%) of LTRs. (*D*) A summary of peaks in LTR elements (sense versus antisense and up versus non-up). Fractions of peaks in other repeats do not show such differences (Fig. 2B; Supplemental S2B–D). For example, SINE elements are 2.0% for up and 2.6%–4.2% for other cases, while SVA elements are 0.1% for up and 0.0%–0.1% for other cases. (*E*) Expression levels of 935 LTRs in tumor, nontumor (NT), and normal liver tissues. “Liver” (normal adult and fetus liver tissues) and “Hepato” (three biological replicates of primary hepatocytes) data from the FANTOM5 Project are shown for comparison. To avoid log-of-zero errors, 0.005 was added to the tpm values for this box plot. The median values of “Normal,” “Liver,” and “Hepato” are 0.005, indicating 0 tpm. (*F*) Activated LTR subfamilies versus copy numbers in the human genome. (*G*) Relative position of CAGE peaks on LTR12C. The majority of CAGE peaks are located at a relative position of 70% from the 5′ end. Black bar and green arrow: presumed promoter architecture conserved among LTR12C elements. (*H*) Distribution of CAGE tags on long (>5 kb) LINE elements. Blue (purple) bars: sense (antisense) direction with respect to the LINE elements. Arrows: potential promoters embedded in full-length L1 elements.

We compared expression levels between a case group of 50 tumors and a control group of 50 matched nontumors using edgeR (Robinson et al. 2010) to explore misactivated transcripts in tumors (see Methods). We identified 14,477 significantly up-regulated peaks in tumors with a low FDR threshold (FDR < 0.05), among which 4942, 4779, and 4756 were coding, proximal, and distal peaks, respectively (Fig. 2A). The most significantly up-regulated protein-coding gene is *GPC3* (Supplemental Fig. S2A), which is a known biomarker and a possible target for the treatment of HCC (Capurro et al. 2003; Gao et al. 2014). About 42% of the top 100 distal peaks overlapped with repetitive elements in the sense direction. To estimate the trend toward association of up-regulated peaks and repetitive elements, we examined which fraction of the up-regulated peaks overlapped with major repetitive elements (LINE, LTR, and SINE). The family names and genomic positions of repetitive elements we used are those defined by RepeatMasker (http://www.repeatmasker.org) and RepBase (Jurka et al. 2005) based on their classification and nomenclature of eukaryotic transposable elements (Kapitonov and Jurka 2008). Interestingly, ∼20% (935/4756) of the up-regulated distal peaks overlapped with LTR elements in the sense direction (Fig. 2B). The fraction of LTRs increases to 30% (127/408) when limited to the most significantly up-regulated peaks with FDR below 1.0 × 10^−8^ (Fig. 2C). These percentages are significantly higher (*P* < 1.0 × 10^−5^ in all cases; one-sided Fisher's exact test) than those found for (1) non-up-regulated peaks (6.3%) (Supplemental Fig. S2B), (2) up-regulated peaks in the antisense direction (7.1%) (Supplemental Fig. S2C), (3) non-up-regulated peaks in the anti-sense direction (7.4%) (Supplemental Fig. S2D), (4) LINE and SINE elements in sense and antisense (1%–7%) (Fig. 2B; Supplemental Fig. S2C), and (5) randomized distal peaks (5%–6%) (Supplemental Fig. S2E). A summary of the main four overlap patterns is shown in Figure 2D. It was also true (1) when we excluded “-int” elements from the LTR category, which are internal elements of ERV (endogenous retrovirus) (Supplemental Fig. S2F), (2) when we used tags with mapping quality 30 or more (Supplemental Fig. S2G), and (3) when we used GENCODE v.19 annotations for our peak classification instead of GENCODE v.12 (Supplemental Fig. S2H). This clearly indicates that the promoters located in LTR elements are widely activated in HCCs.

Next, we studied the expression levels of the 935 activated LTR promoters in 10 normal livers using three data sets: normal liver tissues from this study (*n* = 5) (Supplemental Table S1); adult and fetus liver tissues from FANTOM5 (*n* = 2) (Supplemental Table S2); and primary hepatocytes from FANTOM5 (*n* = 3) (Supplemental Table S2). As expected, the expression levels of LTRs in normal livers are much lower than in nontumor and tumor HCC tissues. More than half of the LTRs were not expressed at all in any of the 10 normal samples (Fig. 2E). We thus conclude that the up-regulation of LTR promoters is a hallmark feature of HCCs. Note that we have confirmed that the non-LTR distal transcripts are also weakly expressed in normal livers (Supplemental Fig. S2I).

About 500 LTR subfamilies with various copy numbers have been defined by RepeatMasker and RepBase for the hg19 human genome. If LTR promoters were randomly activated in unusual conditions such as long-term inflammation, the numbers of up-regulated LTRs should correlate with copy numbers in the genome. If, on the contrary, they were activated through a selective process, one should observe the activation of specific subfamilies, such as LTR7 and HERVH-int, which are markers for pluripotent cells (Santoni et al. 2012; Fort et al. 2014). Figure 2F shows that some subfamilies with relatively small numbers of copies per genome were preferentially activated in HCC tumors compared to the random distribution (Supplemental Fig. S2J), especially the LTR12C subfamily of which 101 elements (104 CAGE peaks) were significantly up-regulated in HCC tumors (Supplemental Table S3). Moreover, the peaks were consistently located at a specific position on the LTR12C elements (Fig. 2G), implying that promoter architecture of LTR elements is conserved. In many cases, the transcription start sites of LTR12C were located within a specific 5-nt (GTGGC) sequence (Supplemental Fig. S2K), whereas nonexpressed LTR12C elements often lost this promoter sequence. Nevertheless, the median size (1411 bp) of 101 up-regulated LTR12C members was similar to the median (1417 bp) of all LTR12C members (Supplemental Fig. S2L), and a subset of nonexpressed LTR12C retained a promoter sequence similar to that of the active elements, indicating that the active and inactive members cannot be distinguished solely by the presence or absence of promoter sequences. Other subfamilies than LTR12C showed enrichment of TSSs around the middle of the elements, which were slightly upstream compared to the position they had in LTR12C (Supplemental Fig. S2M). Conversely, only five elements were up-regulated in the LTR7 and HERVH-int subfamilies, which, it is worth noting, have at least hundreds of elements transcribed in stem cells. In summary, nearly 1000 LTRs were up-regulated in HCC, only a few of which were stem cell–specific LTRs.

The LTRs identified are mostly solitary ones, with a median size of 472 bp, which probably lost their ability to replicate but kept their promoter activity. On the other hand, full-length LINE elements are known to be activated with copy number increase in multiple cancers (Lee et al. 2012). To investigate the activity of full-length LINE elements, we mapped CAGE tags to L1 elements longer than 5 kb. Interestingly, tumor and nontumor HCC samples showed very similar expression patterns, irrespective of the etiology, whereas normal livers had very low expression levels of L1 elements (Fig. 2H; Supplemental Fig. S2N). This is consistent with our previous report that increases in copy number were to be found both in tumor and nontumor areas of HCCs (Shukla et al. 2013), although the multiple steps ranging from expression of full-length LINE elements to their retrotranspositions will need to be validated by further experiments. In addition, the antisense promoters that the L1 elements are known to have were clearly observed around 5′ and 3′ regions (Fig. 2H).

### LTR activation increases as HCC progresses in *Mdr2* knockout mice

**Figure 3.**
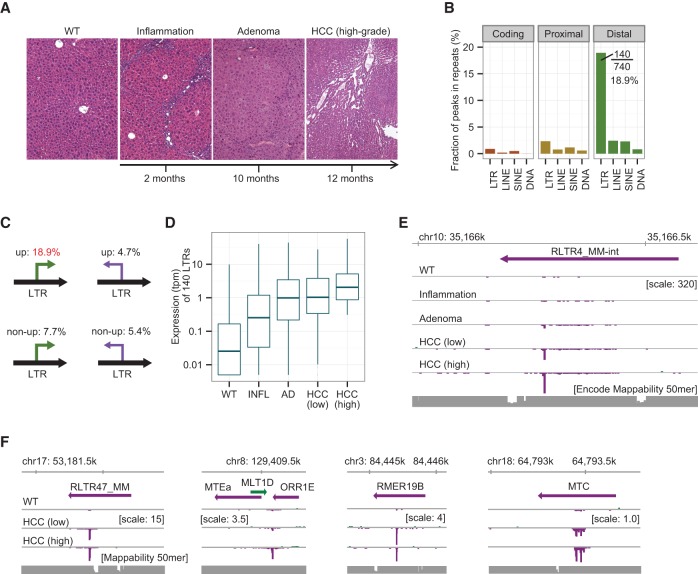
LTR promoters are activated in mouse HCCs. (*A*) Representative liver histological sections at the indicated stages of carcinogenesis in *Mdr2* KO mice. (*B*) Fraction of peaks overlapping with repetitive elements in the sense direction. Nearly 19% (140/740) of up-regulated distal peaks overlap with LTR elements. (*C*) Fractions of peaks overlapping with LTRs according to the (sense versus antisense) direction of transcription and the degree of up-regulation. (*D*) Expression levels of the 140 LTRs in normal (WT), inflammatory (INFL), adenoma (AD), low-grade, and high-grade HCC tissues. The data are means over tumors belonging to a given histological group (tpm + 0.005). (*E*) CAGE signal of the most significantly up-regulated LTR promoter at the indicated stages of the liver disease. The signal increases as the disease develops. CAGE tags are pooled for each group and visualized using the ZENBU Browser. (*F*) Expression patterns of LTRs selected from four different subfamilies.

We previously reported that LTR activation is frequently observed in human and mouse stem cells (Fort et al. 2014). Given that LTR activation occurs in human HCC, as shown above, we assumed that it also occurs in mouse HCC, giving us the opportunity to study LTR activation at different stages of liver carcinogenesis. To this aim, we used the *Mdr2* KO mouse, a well-known model of inflammation-driven HCC (Pikarsky et al. 2004; Iannelli et al. 2014). We examined 37 liver samples from either wild-type (WT) or *Mdr2* KO mice. Liver tissues from *Mdr2* KO mice were categorized into the following four histological groups: inflammation; adenoma; low-grade HCC; or high-grade HCC (>50%) (Fig. 3A; Supplemental Table S4; Katzenellenbogen et al. 2007). We sequenced CAGE libraries for six WT, three inflammation, four adenoma, 16 low-grade HCC, and eight high-grade HCC samples. We determined 49,096 CAGE peaks classified into coding, proximal, and distal groups using the Ensembl annotation (Supplemental Fig. S3A). By comparing the expression levels of WT (*n* = 3) and high-grade HCC (*n* = 4) tissues, we identified 740 significantly up-regulated distal peaks characteristic of high-grade HCC (Supplemental Fig. S3B). As in the case of human HCC, ∼19% (140/740) of the up-regulated distal peaks were located within LTR elements with the same strand (Fig. 3B; Supplemental Table S5), which is a much higher proportion than for the other patterns (Fig. 3C; Supplemental Fig. S3C). It was also true (1) when we excluded “-int” elements from the LTR category and (2) when we used tags with a mapping quality of 30 or more (Supplemental Fig. S3D). These same 140 LTRs were also overexpressed in adenoma and HCC tissues of *Mdr2* KO mice starting from the preneoplastic inflammation stage (Fig. 3D). Figure 3E shows the most significantly up-regulated distal peaks derived from LTRs, the expression levels of which increased as carcinogenesis progressed. The expression patterns of the four different elements selected from noninternal LTR families show consistent promoter activation within LTR elements in low- and high-grade HCC (Fig. 3F). Taken together, these results indicate that activation of LTR promoters is a shared feature of human HCC and the mouse HCC model studied.

### Molecular signature of HCC comprising top 43 up-regulated LTR-derived transcripts

**Figure 4.**
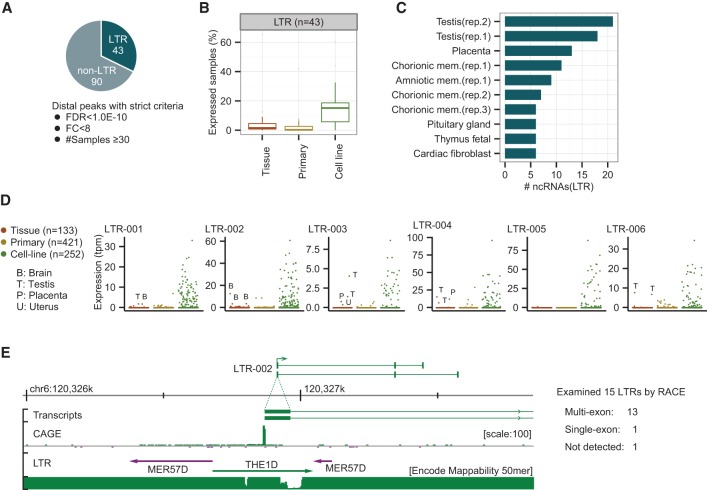
LTR-derived ncRNAs found in HCCs are also expressed in reproductive tissues and cell lines. (*A*) Number of LTR-derived (43) and non-LTR-derived (90) ncRNAs among the up-regulated distal ncRNAs. The selection criteria are as follows: FDR threshold < 1.0 × 10^−10^; fold change > 8; expression in at least 30 tumor samples. (*B*) Proportion of tissues, primary cells, and cell lines that expressed the 43 LTR-derived ncRNAs according to FANTOM5. The LTR expression threshold value is the median expression level of the working set of 50 HCC tumors. (*C*) Top 10 normal tissues and primary cells that express the LTRs according to FANTOM5. (Rep) Biological replicates prepared in FANTOM5. (*D*) Expression patterns of the six most strongly expressed LTRs in the human body. LTR-003, 004, and 006 are only expressed in reproductive tissues (testis, placenta, and/or uterus). LTR-001 and 002 are expressed in brain. (*E*) Full-length ncRNAs with LTR promoters determined by 3′ RACE. Thirteen out of 15 LTRs are confirmed as promoters of multiexon ncRNAs. LTR-002 is shown as an example of identified transcripts.

Having found significantly up-regulated distal peaks with a low (<0.05) FDR threshold in human and mouse HCCs, we sought to select human ncRNA candidates appropriate for determining a molecular signature of HCC using the following stringent criteria: (1) FDR threshold below 1.0 × 10^−10^; (2) fold change above 8.0; and (3) expression in at least 30 tumor samples. About one-third of the distal peaks (43/133) that passed the criteria coincided with LTRs (Fig. 4A). These 43 signature-LTRs were given sequential names from LTR-001 to LTR-043 in ascending order of FDR values. For a full list and the expression patterns, see Supplemental Table S6 and Supplemental Figure S4A. The fact that the signature-LTRs are scarcely transcribed in normal liver tissues raises the question of the nature of the tissues in which they are intrinsically programmed for expression. According to FANTOM5, their expression is limited to 1%–2% of the normal tissues and primary cells (Fig. 4B; Supplemental Fig. S4B). Intriguingly, reproductive tissues and primary cells occupy the top seven positions. Testis, which expresses about half of the 43 signature-LTRs, is in the first position followed by placenta, chorionic, and amniotic membranes (Fig. 4C). Some of the highest-ranking signature-LTRs, such as LTR-003, LTR-004, and LTR-006, are exclusively expressed in reproduction-related tissues (Fig. 4D). In contrast, the top six signature-LTRs are widely detected in all hepatic and nonhepatic cancer cell lines (median: 38 samples) (Fig. 4B,D; Supplemental Fig. S4B), emphasizing the close link between these LTRs and carcinogenesis.

We submitted 15 of the 43 signature-LTRs to 3′ RACE validation (Supplemental Table S7), among which LTR-001 and 002 were further investigated by nested PCRs using 10 more primer pairs designed on different exons (Supplemental Fig. S4C). Deep sequencing of 3′ RACE and PCR products using MiSeq successfully determined the transcript structures of 14 of them, among which 13 had at least one splice site (Supplemental Table S8). The 33 transcripts detected, including splice variants, were all predicted to be ncRNAs with low coding potential by the Coding-Potential Assessment Tool (CPAT) (Supplemental Table S9; Wang et al. 2013). The median exon size is 154 bp, which is comparable to the average coding gene size (122 bp), whereas the median intron size is ∼37 kb, i.e., much longer than the general protein-coding gene size (1 kb) (Lander et al. 2001). Figure 4E shows an example of novel transcripts, LTR-002, determined by the 3′ RACE. The TSS is located at the middle of an LTR element, and the second and third exons are located far downstream from the first exon without encoding any viral proteins, suggesting that LTR-002 has become fixed as a promoter provider to the ncRNA. Note that we also confirmed that seven selected non-LTR candidates are all multiexon ncRNAs by 3′ RACE and CPAT (Supplemental Tables S8, S9).

### Stratification of HCCs based on the activation level of LTR promoters

**Figure 5.**
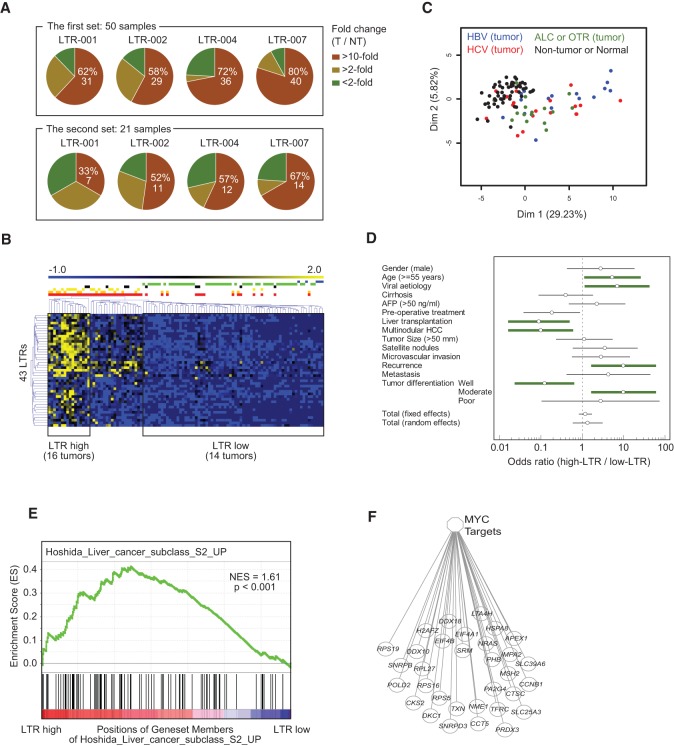
LTR promoters are frequently activated in human HCCs, correlating with viral etiology and aggressiveness. (*A*) Fold changes of LTR-001, 002, 004, and 007 in tumor compared to nontumor samples measured by qPCR in the working (first) and validation (second) set of HCC samples. (*B*) Unsupervised classification of HCC samples according to the expression profile of the 43 signature-LTRs using the MeV (MultiExperiment Viewer) software. Three (LTR-high, LTR-intermediate, LTR-low) distinct clusters are clearly visible. The LTR-high cluster included 16 tumor samples. The LTR-low cluster comprised 14 tumor, 50 NT, and five normal samples. (Blue) Normal liver, (green) NT, (black) alcohol, (yellow) HBV, (orange) HCV, (red) all tumors. (*C*) PCA based on the expression profile of 43 signature-LTRs. (*D*) Correlation between the indicated clinical parameters and the expression level of the signature-LTRs. Thick green lines: clinical features significantly associated with either the LTR-high or the LTR-low subclass according to the criterion FDR < 0.05. (*E*) Gene set enrichment analysis (GSEA) plot of HCCs related to LTR activity. Genes were rank-ordered according to differential expression between LTR-high and LTR-low HCC subclasses. The LTR-high HCC subclass revealed an enrichment of proliferative genes comparable to that of the S2 subclass of Hoshida et al. (2009). The black bars on the *x*-axis represent positions of gene set members on the rank ordered list of gene set Hoshida_liver_cancer_subclass_S2_up. The *y*-axis represents the enrichment score (ES) of the gene set Hoshida_liver_cancer_subclass_S2_up, in the subclass of samples LTR-high HCC. (NES) Normalized Enrichment Score; NES = ES/mean (all ES of other gene sets in MSigDB 4.0). (*F*) Enrichment plot showing an up-regulation of MYC target genes in the LTR-high subclass.

We validated the CAGE data by measuring the expression levels of LTR-001, 002, 004, and 007, which are all multiexon ncRNAs, as determined by 3′ RACE, using quantitative real-time PCR in the previous working set of 50 tumor and matched nontumor (T/NT) HCC samples and in a validation set of 21 new T/NT HCC samples. The primer sequences are listed in Supplemental Table S10. Consistent with the CAGE data, the expression of these LTRs was more than 10-fold higher in ∼60%–80% of the tumor samples compared to the matched NT in the working set (Fig. 5A; Supplemental Fig. S5). An up-regulation of the selected LTRs was also observed in a large majority of the tumors in the validation set. Globally, LTR-007 was the most frequently (76%, 54/71) expressed LTR in HCC tumors. These data show that LTRs are recurrently activated in HCC, with different activation levels for different LTRs and different HCCs, indicating that the LTR activation is not the consequence of genome-wide random events during tumorigenesis. Figure 5B shows an unsupervised classification of the 105 CAGE samples according to the ncRNA expression level of the 43 signature-LTRs. This revealed three (high, moderate, low/inexistent) well-defined clusters of samples. The LTR-high cluster contained only tumor HCC samples (16 in number), while the LTR-low subclass contained all the nontumor and normal-liver samples and 14 tumor samples. Principal component analysis (PCA) showed that LTR-high ncRNA expressions clustered almost exclusively (90%) with viral etiology, the highest expressions occurring mostly in HBV-positive tumors (Fig. 5C). The remaining 10% were related to alcoholism. The tumors of the LTR-low subclass were equally divided between the different etiologies.

A multivariate-analysis based on the set of clinical parameters listed in Figure 5D shows that the HCCs of the LTR-high subclass were globally more severe than those of the LTR-low subclass (Fig. 5D; Supplemental Table S11). The LTR-high HCCs were significantly correlated with age ≥55 yr, viral etiology (as already mentioned), and a high risk of recurrence. Their differentiation grade ranged from moderate to poor. They, moreover, had a clear tendency to be associated with more satellite nodules, more microvascular invasion, and more distant metastasis. LTR-low HCCs were mostly well-differentiated, and more importantly, they had a much lower risk of recurrence (2/14) than the LTR-high ones (10/16; *P* = 0.011). A correlation with patient outcome was irrelevant to our purpose due to the fact that the majority of the patients with LTR-low HCC received a hepatic graft, improving their outcome compared to patients with LTR-high HCC who benefited from a partial hepatectomy.

Finally, to gain insight on the molecular pathways activated in the different HCC subclasses, we performed a gene set enrichment analysis (GSEA) from the CAGE transcriptome. We found a great enrichment of genes related to cell cycle and replication in the LTR-high subclass compared to the LTR-low one (Fig. 5E,F). Interestingly, the gene enrichment pattern of the LTR-high HCCs was closely similar to that of the S2 molecular subclass defined by Hoshida et al. (2009), which is characterized by proliferation as well as *MYC* and *AKT1* activation. These results suggest that the LTR expression level be taken as a new characteristic in the definition of HCC molecular subclasses, corresponding to an as-yet unsuspected layer of complexity in the genomics of HCC.

### Distal ncRNAs activated by cobinding transcription factors

**Figure 6.**
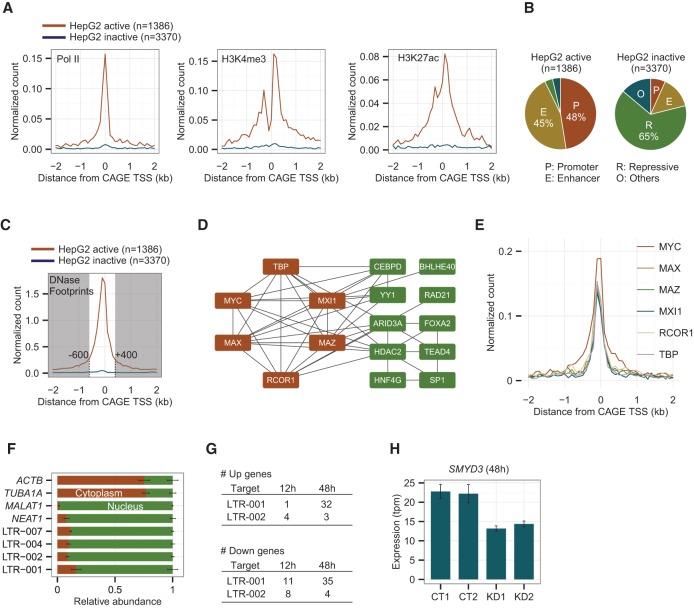
A subset of up-regulated distal ncRNAs including LTRs and non-LTRs has active histone marks and TF binding sites in the HepG2 cell line. (*A*) Enrichment of ChIP-seq peaks for RNA Pol II, H3K4me3, and H3K27ac within 2 kb from the center of the CAGE peaks in 100-bp bins. The red and blue lines represent active and inactive peaks in the HepG2 cell line, respectively. (*B*) Chromatin states annotated by ENCODE based on combinatorial patterns of chromatin marks for the HepG2 active and inactive loci. (*C*) Distribution of DNase I footprints within 2 kb from the center of the CAGE peaks in 100 bp bins. (*D*) The co-occurrence of transcription factors at up-regulated distal ncRNA loci. Nodes are connected by a solid line if their Jaccard Index is above 0.5. Nodes shown in red are fully connected to one another. (*E*) Enrichment of ChIP-seq peaks for the fully connected six TFs within 2 kb from the center of the CAGE peaks in 100-bp bins. (*F*) Relative abundance of four LTR ncRNAs in nucleus and cytoplasm with *ACTB*, *TUBA1A*, *MALAT1*, and *NEAT1* as cytoplasmic and nuclear controls. (*G*) Numbers of significantly up-regulated genes after 12 and 48 h of knocking down LTR-001 and 002. (*H*) Expression levels of *SMYD3* measured by CAGE in CT (control) and KD (knockdown) samples at 48 h of the ASO treatment.

To cast light on the mechanism of up-regulation of ncRNAs, we combined our data for human HCCs with the large set of data for HepG2 cells provided by ENCODE. We first examined whether the up-regulated distal peaks that we found in human HCCs (see Fig. 2A) were also active in HepG2 cells. Of the 4756 up-regulated distal peaks in human HCCs, 1386 had both transcripts and open chromatin signals in HepG2 cells. This observation is further validated by independent ChIP-seq data on RNA polymerase II (Pol II) and active histone marks (H3K4me3 and H3K27ac). Figure 6A shows that these active signals were strongly enriched around HepG2 active peaks, whereas none was enriched around HepG2 inactive peaks. Furthermore, the former loci were mostly marked as promoters or enhancers, while the latter were marked as repressive states according to chromatin state annotation based on combinatorial patterns of chromatin marks (Fig. 6B; Ernst et al. 2011). We thus conclude that 1386 distal peaks were commonly active both in HCC tumor tissues and the HepG2 cell line. Subsequently, we used only these active peaks for further analyses. The list of ENCODE data (CAGE, DNase-seq, FAIRE-seq, ChIP-seq, and chromatin states) used to assess the activities of the distal peaks in HepG2 cells is available in Supplemental Table S12.

The analysis of the promoter region around the 1386 distal peaks active in HepG2 cells revealed that distal ncRNAs carry multiple binding sites of various transcription factors in distinct positions. The DNase I footprint represents direct binding loci of regulatory elements including transcription factors. DNase I footprint data showed that the region spanning from −600 to +400 bp (local regulatory region) around the active peaks was highly enriched in transcription factor binding sites, with five or more footprints at more than half of the peaks (Fig. 6C). We then searched for the transcription factors that contributed to up-regulation of ncRNAs using the ENCODE ChIP-seq data of HepG2 for 51 transcription factors (Supplemental Table S13). Interestingly, MYC, an oncogene which is widely activated in many cancers, bound to ∼45% (635/1386) of the active peaks, while it bound to a significantly smaller proportion of the non-up-regulated peaks (one-sided Fisher's exact test with Bonferroni correction < 0.05). In total, 19 transcription factors were significantly associated with up-regulated peaks, including TBP (TATA box binding protein), which had the lowest *P*-value, and ARID3A (AT-rich interactive domain-containing protein 3), which had the highest binding counts (Supplemental Fig. S6A). We then investigated which pairs of TFs shared common targets using the Jaccard index. More specifically, we generated a cobinding network, in which each node represents a transcription factor and each edge represents a cobinding pair, whose Jaccard Index is above 0.5 (Fig. 6D). Six transcription factors in the network are fully connected to one another, and four of them include MYC, the MYC-associated protein MAZ, the MYC dimerization partner MAX, and the alternative MAX partner MXI1. Moreover, all six transcription factors show binding immediately upstream of TSSs (between −100 and 0 bp), suggesting an active role for these transcription factor complexes in the deregulation of ncRNAs in HCC (Fig. 6E). In addition, we performed similar analyses for LTR-derived distal peaks, which is a subset of the 1386 peaks analyzed above, and observed similar enrichment patterns of RNA Pol II, active histone marks, DNase footprints, and six transcription factors (Supplemental Fig. S6B,C).

We finally performed knockdown experiments to investigate whether LTR-derived ncRNAs have any functional role in the regulatory network of HepG2 cells. Because recurrently activated LTR-derived ncRNAs are enriched in the nuclear fraction of HepG2 cells (Fig. 6F), we designed locked nucleic acid (LNA) oligonucleotides (Petersen and Wengel 2003) for the top two LTR-derived ncRNAs. LNAs were designed on the common exon of all isoforms. LTR-001 and LTR-002 were stably suppressed by two different LNAs per target (Supplemental Table S14) at 12, 24, 48, and 72 h after transfection into HepG2 cells (Supplemental Fig. S6D). We selected the 12- and 48-h time points to study early responses to the depletion of LTR-001 and LTR-002 by CAGE analysis (Supplemental Fig. S6E). We confirmed that the transcriptome of cells treated by two different LNAs were highly correlated (Supplemental Fig. S6F). A total of 98 differentially expressed genes (FDR < 0.05) were identified by comparison between knockdown and control samples using edgeR (Fig. 6G). Supplemental Tables S15 and S16 display the full list of the dysregulated genes for LTR-001 and LTR-002 at 48 h. Among the down-regulated genes with LTR-001 stands *SMYD3*, which encodes a lysine methyltransferase involved in the methylation of MAP3K2, increasing MAP kinase signaling and promoting the formation of Ras-driven carcinomas (Hamamoto et al. 2004; Mazur et al. 2014). We found that the expression level of *SMYD3* was unchanged at 12 h but significantly lowered at 48 h (Fig. 6H). However, the protein level of SMYD3, expression levels of MAP kinase target genes, and cell proliferation were not significantly changed, suggesting that a knockdown of LTR-001 is not enough to disturb the downstream pathways of SMYD3. Among the up-regulated genes, *IGFBP1* (insulin-like growth factor binding protein 1) has important inhibitory role in the development and/or growth of HCC, and *RBBP6* (retinoblastoma binding protein) acts as a cell-cycle regulator.

## Discussion

A growing number of ncRNAs are being considered as regulators of various cellular processes (Mercer and Mattick 2013), whereas relatively few ncRNAs are reported to be associated with hepatocarcinogenesis (Braconi et al. 2011; Spizzo et al. 2012). We have established a comprehensive map of promoters for ncRNAs actively transcribed in a large set of human HCCs as well as in the *Mdr2* KO mouse model of HCC using CAGE. This unique resource provides a mammalian promoter map for liver cancer at single-nucleotide resolution, which enables us to connect promoter activities with surrounding DNA regulatory elements, including LTR retroviral promoters and transcription factor binding sites. The comparison of the expression levels between tumors and matched nontumor tissues revealed that more than 14,000 promoters are significantly up-regulated in HCC tumors, one-third of which are at least 5 kb away from the promoters of known protein coding genes. Remarkably, 20% (935/4756) of the up-regulated distal peaks are LTR retroviral promoters, which are mostly silenced in normal hepatocytes and liver tissues. Nevertheless, a subset of the LTRs is strongly expressed in reproduction tissues, such as testis and placenta, indicating a highly tissue-specific regulation of the LTRs. The mechanisms of regulation of the LTRs was previously investigated in iPS and ES cells, which express high levels of specific LTR families, namely, LTR7 and HERVH. An appropriate activation of these LTRs was shown to be essential for iPS reprogramming (Lu et al. 2014; Ohnuki et al. 2014). Depleting LTRs in ES cells led to cell differentiation (Fort et al. 2014; Lu et al. 2014), whereas augmenting LTRs impaired cell differentiation (Ohnuki et al. 2014). Concordantly, we found that HCCs can be stratified according to LTR expression level and that LTR-high HCCs are less well-differentiated than LTR-low ones, confirming that an excess of LTR activity impairs cell differentiation. It is interesting that nonorthologous LTR subfamilies are activated in both human and mouse HCCs. The same type of unexpected concordance was previously observed in stem cell-associated LTRs (Fort et al. 2014). Whether or not this should be attributed to convergent evolution of functionally similar features will remain uncertain as long as LTR-related functional pathways are not determined.

In this study, we used tags uniquely mapped to the genomes. This strategy might miss transcripts derived from young ERV elements that accumulate few mutations. To assess the potential impact of multimap tags that mapped to multiple genomic loci (mapping quality < 10), we have performed analyses of repetitive elements at the family level. We measured expression values for each repeat family defined by RepeatMasker in the human and mouse genomes based on all tags, including multimap tags, and compared tumors with nontumors using edgeR. We confirmed that various LTR families are significantly up-regulated in tumors, whereas most young ERV elements, in particular, human ERVK (LTR5_Hs), SVA, *Alu*, and mouse IAP elements, are not up-regulated or are even down-regulated in tumors (Supplemental Figs. S2O, S3E). This shows that young repetitive elements are not largely activated in liver cancer.

This study provides evidence supporting the idea that activation of LTR promoters might contribute to liver carcinogenesis. First, a subset of 43 LTRs was up-regulated (more than eightfold) in the vast majority of the HCCs studied. For example, LTR-007, which was totally suppressed in normal liver and most other healthy tissues, was more than 10-fold up-regulated in ∼80% of the HCC tumors. This high frequency of LTR-007 activation is comparable to the frequencies of the *GPC3* activation (Jia et al. 2007) and *TP53* mutations in HCC (Fujimoto et al. 2012). In addition, LTR-007 is up-regulated more than 1000-fold in 15 patients from the initial data set and seven patients from the second data set. Up-regulation rates of this magnitude are likely due to promoter activations rather than copy-number increase. Second, LTR-high tumors showed a higher risk of recurrence and, more generally, were more aggressive than LTR-low ones. The LTR-high subclass was closely similar to the well-recognized S2 molecular subclass of HCCs (Hoshida et al. 2009), suggesting taking into account the LTR promoter activity in the taxonomy of HCCs. Third, the LTRs found in HCC tumors were expressed in several cancer cell lines. Fourth, LTR up-regulation appeared from the preneoplastic stage in the *Mdr2* KO mouse model of HCC. This study uncovers a new layer of complexity in the transcriptome of HCC, which highlights the importance of understanding LTR-derived ncRNAs in cancer. The next stage would be the functional characterization of the ncRNAs, which requires a series of experiments including overexpression, knockdown, and/or knockout. The transcriptional responses of cell lines and HCC tissues are different, and the best ncRNA candidates found in HCC tissues might not be testable using cell lines, suggesting that a much larger number would be needed to find ncRNAs that are equally significant in function.

## Methods

### CAGE libraries for human liver tissues

Liver tissues were collected from 50 patients resected for HCC and five patients resected for metastatic liver colon cancer. The latter were used as control samples. The ethics evaluation committee of the Inserm (IRB00003888, FWA00005831) and the ethical review committee of RIKEN (H24-4) approved the use of human liver samples in this study. Informed consent was obtained from each subject. Total RNAs were extracted from the tissues using a mirVana kit (Ambion). The quality of the RNA samples was assessed using an Agilent RNA 6000 Nano kit (RIN scores provided in Supplemental Table S1). We prepared CAGE libraries following the protocol described in detail in Takahashi et al. (2012). We used 5 µg of total RNA to synthesize cDNA with random primers. Full-length cDNAs were biotinylated and captured by streptavidin-coated magnetic beads. The cDNAs were released from cap-trapped RNAs, ligated to 5′ linkers including barcode sequences, and digested with EcoP15I. CAGE libraries were sequenced with single-end reads of 50 bp on the Illumina HiSeq 2000 platform.

### Determination of CAGE TSSs

Multiplexed sequencing reads were split by barcode sequences. Reads with ambiguous bases “N” were removed, and linker sequences were trimmed from the 3′ end. Artificial sequences and ribosomal RNAs were identified and removed using TagDust (Lassmann et al. 2009) and RNAdust (http://compbio.gsc.riken.jp/rnadust.html). We then aligned the extracted CAGE tags to the human genome (hg19/GRCh37 assembly) using BWA v0.5.9 (Li and Durbin 2009) with default parameters on the MOIRAI pipeline platform (Hasegawa et al. 2014). The uniquely mapped CAGE tags with a minimal mapping quality 10 were used in this study. Because the most 5′ position of a CAGE tag represents a TSS, the 5′ coordinates were extracted from the tags and generated a genome-wide TSS map at single-nucleotide resolution. We then clustered the tags to define distinct CAGE peaks using Paraclu with the following parameters: (1) a minimum of 10 tags per cluster; (2) a minimum density increase of 2; and (3) a maximum cluster length of 500 bp. Raw tag counts for each peak were divided by a total tag count of the library to calculate normalized expression values. The unit of the expression value is tpm, tags per uniquely mapped million tags. CAGE tags and peaks were visualized using ZENBU (Severin et al. 2014).

### Identification of differentially expressed peaks

The differentially expressed peaks were identified using edgeR version 2.6.3 (Robinson et al. 2010) in a Bioconductor package in R. Briefly, the dispersion of expression values was estimated by the quantile-adjusted conditional maximum likelihood (qCML) method using estimateCommonDisp and estimateTagwiseDisp functions with default parameters. Exact *P*-values and false discovery rates were calculated using exactTest and topTags functions.

### *Mdr2* KO mouse and CAGE libraries

Animal experiments have been performed in agreement with the Italian Laws (D.L.vo 116/92) and the guidelines of the European Commission Recommendation 2007/526/EC-June 18, 2007. The project has been reported to the Italian Ministry of Health (project n. 106/11). Founders of the FVB.129P2-Abcb4tm1Bor/J (*Mdr2* KO, stock number: 002539) and FVB/NJ (*Mdr2* WT, stock number: 001800) mice were purchased from the Jackson Laboratory. Mouse colonies were maintained in a specific pathogen-free animal facility. Tumor growth in *Mdr2* KO livers is multinodular, and HCC tends to develop within an adenoma (as a focus of tumor progression). The histological composition of grossly detectable hepatic nodules was semiquantitatively determined based on reported classification criteria (Thoolen et al. 2010). In order to analyze sample sets based on histological content, we classified samples as “adenoma” when scoring a variable content in adenomatous tissue without any detectable presence of carcinoma, “low-grade HCC” with a histologic content of carcinoma ≤ 50%, and “high-grade HCC” when the carcinoma content was higher than 50%. The histological composition of the liver tissues was determined based on the criteria reported in the literature (Katzenellenbogen et al. 2007).

We prepared and sequenced CAGE libraries from control and tumor tissues as described above. The sequences were aligned to the mouse genome (mm9/NCBI37 assembly) using BWA v0.5.9 (Li and Durbin 2009) with default parameters. The uniquely mapped CAGE tags with a minimal mapping quality 10 were used for the downstream analysis. CAGE peaks were detected using the Paraclu code.

### Comparative analysis with FANTOM5 data

We calculated expression values of the 43 LTRs in various cell types using FANTOM5 expression data (The FANTOM Consortium et al. 2014). More specifically, the number of tags within a CAGE peak was counted and normalized by the library size with mapping quality ≥ 20 using the files in the ctss.bed format provided in the FANTOM5 web site (http://fantom.gsc.riken.jp/5/datafiles/latest/basic/). This process was done for the CAGE libraries of 135 tissues, 432 primary cells, and 241 cell lines (the full sample list is available in Supplemental Table 1 of the FANTOM5 paper [The FANTOM Consortium et al. 2014]). To see which cell types express the LTRs, expression values of the LTRs in the FANTOM5 samples were compared to the expression values of the tumors. The number of samples that express the LTRs with more than the median values of 50 liver tumors was counted for tissue, primary cell, and cell line groups. We also counted the number of LTRs that express in each FANTOM5 sample.

### Sample clustering based on the LTR expression

The graphical representation of CAGE LTR expression profile and unsupervised classification of HCC samples were performed with the MeV (MultiExperiment Viewer) software, version 4.9.0 using the Euclidean distance metric with average linkage algorithm during classification (Saeed et al. 2003). The obtained cluster allowed discrimination of two classes of tumor samples: tumors with low LTR expression and tumors with high LTR expression.

### Multivariate analysis on clinical biological parameters

The detection of LTR level expression allowed us to distinguish two groups of tumor samples by CAGE analysis (high level and low level of LTR discriminated by unsupervised clustering). The odds ratio of each clinical and biological criterion was calculated for each class of patients (high-LTR and low-LTR). Meta-analysis performed with the totality of clinical and biological criteria taken into account during the study allowed testing of the heterogeneity of the calculated odds ratios. A forest plot was drawn with odds ratios and interval of confidence at 95% for each criterion. Significance of the meta-analysis was retained if the *P*-value of the test for heterogeneity < 0.05 and if the random effects on the forest plot are well centered.

### Comparative analysis with ENCODE HepG2 data

We used CAGE, DNase-seq, and FAIRE-seq data of the HepG2 cell line, downloaded from the ENCODE website (http://genome.ucsc.edu/ENCODE/) to determine which tumor TSSs are active in HepG2. First, all CAGE tags produced for different compartments (Supplemental Table S12, No.1–9) were combined. Secondly, narrow peaks, defined by the ENCODE pipeline, of DNase-seq rep1, rep2, and FAIRE-seq (Supplemental Table S12, No.10–12) were combined. Finally, tumor TSSs were determined to be active in HepG2 if at least one CAGE tag was found in the TSS and at least one narrow peak of the open chromatin signals was found within 1 kb of the TSS.

We used chromatin states systematically annotated based on combinatorial patterns of chromatin marks (Supplemental Table S12, No.16; Ernst et al. 2011) to examine epigenetic differences in active and inactive TSS loci. The original 15 categories were simplified into four categories, in which active, weak, and poised promoters were merged as “Promoter,” strong and weak enhancers were merged as “Enhancer,” repressed and heterochromatin were merged as “Repressive,” and all the others were merged as “Others.” Each TSS was annotated to one of these categories. When a TSS is overlapped with multiple segments, the annotation was prioritized in the order of Promoter, Enhancer, and Repressive.

We used ChIP-seq peaks, defined by the ENCODE pipeline, for 51 transcription factors (Supplemental Table S13) to test associations between TF bindings and up-regulations. For each TF, a 2 × 2 contingency table was generated in which one factor is whether or not distal TSSs are significantly up-regulated and the other factor is whether the TSSs have a binding site of the TF (midpoint of the peak) in a range from −600 to +400 bp. A *P*-value was calculated for each TF using a one-sided Fisher's exact test and adjusted for multiple testing using Bonferroni's correction. A Jaccard index was calculated for all pairs of significantly associated TFs.

### LNA knockdown

HepG2 cells were maintained in Dulbecco's modified Eagle's medium, 10% FBS, and 100 units/mL penicillin streptomycin (Gibco) at 37°C, 5% CO_2_. For the qPCR experiment, 80,000 cells were seeded in 12-well plates, and for the CAGE experiment, 160,000 cells were used in 6-well plates. Transfection was done according to the Lipofectamine RNAiMAX protocol. We used LNA longRNA GapmeRs by Exiqon, which are antisense oligonucleotides (ASOs) containing a central stretch of DNA monomers flanked by LNA blocks. Target RNAs are cleaved by RNase H, activated by ASOs. The ASOs were transfected into HepG2 cells at a concentration of 20 nM. Lipofectamine alone and scramble ASOs were used as controls. Knockdown efficiency was measured by qPCR with specific primer sets (Supplemental Table S10) for biological triplicates at 12, 24, 48, and 72 h after the transfection. *GAPDH* was used for normalization.

## Data access

CAGE data from this study have been submitted to the NCBI database of Genotypes and Phenotypes (dbGaP; http://www.ncbi.nlm.nih.gov/gap/) under accession number phs000885.v1.p1 for human HCC and the Gene Expression Omnibus (GEO; http://www.ncbi.nlm.nih.gov/geo/) under accession number GSE60982 for mouse HCC.
